# The effect of additional facet joint block for analgesia in patients with thoracolumbar compression fracture undergoing percutaneous kyphoplasty surgery: A protocol for systematic review and meta-analysis

**DOI:** 10.1097/MD.0000000000029034

**Published:** 2022-03-11

**Authors:** Rongmin Xu, Shundong Li, Guojun Chen, Xin Fan

**Affiliations:** Department of Orthopedics, Taizhou Traditional Chinese Medicine Hospital, Zhejiang, China.

**Keywords:** meta-analysis, pain control, percutaneous kyphoplasty, protocol, thoracolumbar compression fractures

## Abstract

**Background:**

There is currently no pooled data in the literature to support whether additional facet joint block results in better clinical analgesia after percutaneous kyphoplasty. We assessed the existing evidence on the safety and efficacy of facet joint block in the treatment of patients with thoracolumbar compression fractures undergoing percutaneous kyphoplasty based on qualified trials.

**Methods:**

We will search PubMed, Springer, ScienceDirect, Wanfang, and Cochrane Library databases through April, 2022. Cohort studies focusing on assessing and comparing the effect of facet joint block and control group will be included. The studies are screened and evaluated by 2 reviewers independently for eligibility. The following outcome measures must be showed: pain scores, Oswestry Disability Index, satisfaction, and complications observed within both groups from baseline to the end of follow-up period. Review Manager software (v 5.3; Cochrane Collaboration) is used for the meta-analysis. A *P* value of <.05 is considered to be statistically significant. Two independent reviewers will assess the risk of bias of the included studies at study level.

**Results:**

It is hypothesized that additional facet joint block is associated with better pain control.

**Conclusions:**

This study expects to provide credible and scientific evidence for the efficacy and safety of facet joint block in the treatment of patients with thoracolumbar compression fractures undergoing percutaneous kyphoplasty.

**Registration number:**

10.17605/OSF.IO/ARY3C.

## 1 Introduction

The incidence and severity of thoracolumbar compression fractures (TCF) has been steadily increasing in older patients. In the United States, approximately 750,000 adults suffer from TCF each year, with 8 percent of women over age 50 and 27 percent of men and women over age 65.^[1,2]^ TCF occurs due to insufficient anterior vertebral body height and result in spinal deformity, decreased lung function, limitation of abdominal and thoracic contents, impaired mobility, and depression.^[3]^ Conservative treatments such as bed rest, analgesics, and nerve block can reduce pain, stabilize the vertebrae, and restore spinal mobility to some extent. However, conservative treatment may be at risk of poor pain relief, vertebral collapse and kyphosis formation.^[4–6]^ Prolonged bed stay can also lead to subsequent demineralization, increased osteoporosis, bedsores, pneumonia, and deep venous thrombosis.

The percutaneous kyphoplasty described by Garfin et al has been clinically proven in numerous studies to be an effective treatment for rapid pain relief, correction of kyphosis and spinal stability in patients with persistent and severe acute TCF.^[7]^ However, some scholars have noted the phenomenon of residual back pain after percutaneous kyphoplasty. The facet joints have been recognized as a source of low back pain, and injection of local anesthetics (with or without steroids) to block innervation branches has been used to treat chronic low back pain caused by TCF.^[8–10]^ However, it is controversial whether additional facet joint block can be used to relieve pain after percutaneous kyphoplasty.

There is currently no pooled data in the literature to support whether additional facet joint block results in better clinical analgesia after percutaneous kyphoplasty. Therefore, in this meta-analysis, we assessed the existing evidence on the safety and efficacy of facet joint block in the treatment of patients with TCF undergoing percutaneous kyphoplasty based on qualified trials. It is hypothesized that additional facet joint block is associated with better pain control.

## 2 Materials and methods

### 2.1 Study registration

The systematic review protocol has been registered on Open Science Framework registries. The registration number is 10.17605/OSF.IO/ARY3C. The systematic literature review is structured to adhere to PRISMA guidelines (Preferred Reporting Items for Systematic Reviews and Meta-analyses), which include requirements deemed essential for the transparent reporting of results.^[11]^ Ethical approval and patient consent are not required because this study is a literature-based study. We will update our protocol for any changes in the entire research process if needed.

### 2.2 Data sources and search strategy

We will search PubMed, Springer, ScienceDirect, Wanfang, and Cochrane Library databases through April, 2022. Search algorithm are identified as follows: (kyphoplasty) OR (vertebroplasty) AND (compression fracture) AND (facet joint block). The literature search, data extraction, and quality assessments are conducted independently by 2 reviewers. We also search references cited in all included articles to avoid missing other relevant articles. If the effective data are not included in the original articles, we will contact the authors to get them. The studies are screened and evaluated by 2 reviewers independently for eligibility. None of the studies will be excluded due to language restrictions.

### 2.3 Eligibility criteria

We identify literature that met the following inclusion criteria:

1. patients with TCF undergoing percutaneous kyphoplasty surgery;
2. studies focusing on assessing and comparing the effect of facet joint block and control group;
3. the following outcome measures must be showed: pain scores, Oswestry Disability Index, satisfaction, and complications observed within both groups from baseline to the end of follow-up period.

The exclusion criteria are as following:

1. no comparison of facet joint block and control group;
2. abstracts, case reports, letters, conference articles, repeated studies, biochemical trials, meta-analyses, and reviews;
3. lack of useful data in outcomes mentioned above.

### 2.4 Data extraction

Data are extracted by review of each study for population, mean age, gender, follow-up duration, study design, publishing date, characteristics, and outcomes assessment. The 2 reviewers create a study-specific speadsheet in Excel (Microsoft Corp., USA) for data collection. Data extraction will be performed independently, and any conflict is resolved before final analysis. Any disagreements between the 2 reviewers will be discussed and, if necessary, the third author is referred to for arbitration. If the data are missing or can not be extracted directly, authors will be contacted by email. Otherwise, we calculate them with the guideline of Cochrane Handbook for Systematic Reviews of Interventions 5.1.0. If necessary, we will abandon the extraction of incomplete data.

### 2.5 Statistical analysis

Review Manager software (v 5.3; Cochrane Collaboration) is used for the meta-analysis. Extracted data are entered into Review Manager by the first independent author and checked by the second independent author. Risk ratio with a 95% confidence interval or standardized mean difference with 95% CI are assessed for dichotomous outcomes or continuous outcomes, respectively. The heterogeneity is assessed by using the *Q* test and *I*^2^ statistic. An *I*^2^ value of <25% is chosen to represent low heterogeneity and an *I*^2^ value of >75% to indicate high heterogeneity. All outcomes are pooled on random-effect model. A *P* value of <.05 is considered to be statistically significant.

### 2.6 Quality assessment

The Cochrane risk of bias tool will be independently used to evaluate the risk of bias of included randomized cohort studies by 2 reviewers.^[12]^ The quality will be assessed by using following 7 items: random sequence generation, allocation concealment, blinding of participants and personnel, blinding of outcome assessment, incomplete outcome data, selective reporting, and other bias. A modified version of the Downs and Black tool is adopted to evaluate the quality of nonrandomized cohort studies. The modified version consists of 27 items with a total possible score of 29. A score of ≥75% indicates high quality, 60% to 74% indicates moderate quality and ≤60% low quality.^[13]^ Two investigators independently evaluate included studies on the 27 criteria, with any discrepancies resolved by a third independent reviewer. Kappa values are used to measure the degree of agreement between the 2 authors and are rated as follows: fair, 0.40 to 0.59; good, 0.60 to 0.74; and excellent, 0.75 or more.

## 3 Discussion

The primary treatment for TCF consists of conservative methods including bed rest, analgesics, and early rehabilitation with a brace after symptomatic relief. However, a few patients may still complain of severe pain after conservative treatments and even show the progressive collapse of the vertebral body and kyphosis with or without neurological deficit. The percutaneous kyphoplasty described by Garfin et al has been clinically proven in numerous studies to be an effective treatment for rapid pain relief, correction of kyphosis and spinal stability in patients with persistent and severe acute TCF. There is currently no meta-analysis in the literature to support whether additional facet joint block results in better clinical analgesia after percutaneous kyphoplasty. Therefore, in this meta-analysis, we assessed the existing evidence on the safety and efficacy of facet joint block in the treatment of patients with TCF undergoing percutaneous kyphoplasty based on qualified trials. It is hypothesized that additional facet joint block is associated with better pain control.

## Author contributions

**Conceptualization:** Guojun Chen.

**Data curation:** Rongmin Xu, Shundong Li.

**Formal analysis:** Rongmin Xu, Shundong Li.

**Funding acquisition:** Shundong Li.

**Investigation:** Rongmin Xu, Shundong Li.

**Methodology:** Guojun Chen.

**Project administration:** Xin Fan.

**Resources:** Shundong Li.

**Supervision:** Xin Fan.

**Validation:** Xin Fan.

**Visualization:** Xin Fan.

**Writing – original draft:** Rongmin Xu, Shundong Li.

**Writing – review & editing:** Xin Fan.
